# Fear of Evaluation and Online Self-Disclosure on WeChat: Moderating Effects of Protective Face Orientation

**DOI:** 10.3389/fpsyg.2021.530722

**Published:** 2021-08-25

**Authors:** Runxi Zeng, Di Zhu

**Affiliations:** School of Journalism and Communication, Chongqing University, Chongqing, China

**Keywords:** fear of positive evaluation, fear of negative evaluation, online self-disclosure, protective face orientation, WeChat, Chinese context

## Abstract

Fear of evaluation is a key factor that affects how social media users present themselves to others, but little is known about the effects and mechanisms involved, especially on the relationship between fear of positive evaluation and online self-disclosure. This study explores how fear of evaluation affects online self-disclosure and examines how this relationship is moderated by protective face orientation in the Chinese context. A total of 750 Chinese WeChat users constituted the sample for a questionnaire-based analysis and regression analysis. The results showed that both fear of positive evaluation and fear of negative evaluation had a significant negative effect on the amount of online self-disclosure and a significant positive effect on the depth of online self-disclosure. Protective face orientation had a moderating effect on the relationship between fear of evaluation and online self-disclosure for both the amount and depth of online self-disclosure. Our findings suggest that social network site (SNS) users' fear of evaluation can be attributed to their cognitive attitude toward the external environment, and the loss of face in the Chinese context can be included in the social context.

## Introduction

Online self-disclosure refers to the behavior of disclosing personal and private thoughts or feelings in cyberspace in the hope of attracting others' attention or obtaining positive feedback (Krasnova et al., 2010; Valkenburg and Peter, 2011; Chen et al., 2018). Given its easily manipulated, low-cost interaction across space and time, online self-disclosure is becoming a primary platform for daily communication and increasingly occurs on social network sites (SNSs) (Huang, 2016; Wang et al., 2018). However, recent studies have observed the gradual emergence of social avoidance behaviors, such as individuals' reluctance to disclose, non-disclosure or reduced frequency of online self-disclosure (Ketay et al., 2018; Chen et al., 2019). Previous studies have indicated that self-esteem, adult attachment, social anxiety, social isolation, privacy concerns, role stress, and self-concealment may contribute to online self-disclosure avoidance behaviors (Gibbs et al., 2010; Rui and Stefanone, 2013; Hollenbaugh and Ferris, 2014; Lee and Cho, 2018; Chen et al., 2019; Kamalou et al., 2019; Zhang et al., 2019).

The unique technological affordances of SNSs, especially their high levels of visibility and persistence, may increase the potential harm from evaluation by others (Rui and Stefanone, 2018). Therefore, excessive attention to others' opinions and evaluations has become a common factor influencing individual online self-disclosure. Individuals who fear others' evaluation tend to think that others will not view them positively after online self-disclosure, and they may be less willing to reveal themselves on SNSs (Cameron et al., 2009; Rui and Stefanone, 2013). For instance, Long and Neff (2018) found that when individuals reveal themselves in public or fail to meet their ideal expectations, they experience frustration and a reduce sense of self-identity. Although most research has investigated social fear in relation to the unwillingness to self-disclose, few studies have explored the barriers to online self-disclosure, including the fear of rejection, criticism, disapproval and other negative evaluations (Geisler, 2016; Lee and Jang, 2019). Nevertheless, these studies have neglected the possibility that fear of positive evaluation may also influence online self-disclosure behavior.

Moreover, previous studies have suggested that the effects of face on individuals' online self-disclosure differ based on cultural background (Oetzel et al., 2008). Although Chinese face-saving behavior is a common phenomenon, little is known about this behavior by non-Chinese society (Hwang, 2006; Qi, 2011). Specifically, in the Chinese context, an individual with protective face orientation (PFO) is very conscious of others' evaluation at all times and in all places (Hu, 1944; Ho, 1976; Hwang, 2006). To save face, people may selectively present information that is beneficial to their image and identity. If the evaluated cognitive psychology is regarded as a threat of face erosion, Chinese people with a strong PFO will fear losing face as a result of others' evaluations (Chou, 1996; Lee, 2014). However, little is known about how PFO affects the relationship between fear of evaluation and online self-disclosure.

The purpose of this study is to examine the relationship between fear of evaluation and online self-disclosure and the moderating effect of PFO. The data were gathered through a survey of 750 Chinese WeChat users and examined using regression analysis.

## Literature Review

### Fear of Evaluation and Online Self-Disclosure

#### Fear of Evaluation in Social Interaction

Fear of evaluation refers to the social anxiety caused by the evaluation of others, which can be categorized into fear of positive evaluation (FPE) and fear of negative evaluation (FNE) (Weeks et al., 2008; Birk et al., 2019). Initial research concerning the fear of evaluation mainly focused on FNE with the understanding that only negative evaluations by others lead to individual social anxiety and threaten an individual's image (Lombardo and Fantasia, 1976; Heimberg et al., 1988; Valkenburg et al., 2006). As a result, when people receive negative or other unwanted comments that are contrary to their expectations, they may develop fear of evaluation.

Since the proposal of the concept of FPE, most research concerning the fear of evaluation has been based on the two concepts simultaneously (Long and Neff, 2018). FPE refers to the fear of being positively evaluated. The underlying concern is that the individual will not meet social standards and expectations sufficiently or consistently. Moreover, FPE is regarded as a distasteful stimulus by some individuals, and an expected favorable evaluation may eventually become a negative evaluation (Li and Lin, 2016). This change from positive expectation to negative evaluation results in setbacks and social pressure for individuals (Weeks, 2014). People who know that they will be observed and evaluated by a wide audience are more cautious about exposing themselves on social media, thereby reducing their activeness and their enthusiasm for updating dynamics in social space. The FPE proposal helps to improve people's understanding of the general fear of evaluation (Zahavi et al., 2018). Similar to FNE, FPE involves fear of the consequences of positive evaluation (Watson and Friend, 1969; Weeks and Howell, 2012), which induces panic caused by the overaffirmation or expectations of others.

#### Online Self-Disclosure and Social Strategy

The amount of online self-disclosure (AOSD) refers to the frequency and duration of information disclosed on SNSs, and the depth of online self-disclosure (DOSD) refers to the intimacy of the information disclosed on SNSs (Wheeless and Grotz, 1977; Omarzu, 2000; Huang, 2016). Users are accustomed to measuring the success of self-image construction by predicting or perceiving the evaluations of others to determine how to disclose themselves (Cameron et al., 2009; Leary and Allen, 2011; Lee and Jang, 2019). Driven by fear of evaluation, it is a common disclosure strategy to ease social pressure by adjusting one's online self-disclosure habits (Shafique et al., 2017; Zeng and Zhu, 2019; Zhang et al., 2019); especially when the evaluation of others becomes an important reference source of self-recognition, individuals with fear of evaluation tend to magnify their own defects in appearance, comments and behavior from the perspective of being watched and then may fall into a state of social anxiety (Lombardo and Fantasia, 1976; Heimberg et al., 1988). In response to uncertain social risks that may result in disappointment or hurt in interpersonal communication, reducing self-disclosure is a possible strategy to hide defects and maintain self-image (Lee, 2014; Proudfoot et al., 2018; Kamalou et al., 2019; Lin, 2019).

Individuals with fear of negative evaluation are afraid of being blamed, criticized, or ridiculed, sarcasm, and receiving other negative feedback that may damage their personal image, status and even self-confidence (Hwang et al., 2019), resulting in social avoidance (Lombardo and Fantasia, 1976; Heimberg et al., 1988). Such individuals might reduce the number of on-screen appearances or extend the interval between self-disclosures. Individuals with fear of positive evaluation are afraid of accepting others' excessive praise and expectations (Watson and Friend, 1969; Weeks, 2014), producing social avoidance psychology and may choose to reduce the frequency and duration of their SNS disclosures to avoid some praise. Therefore, due to their negative beliefs and emotional experiences of social interactions, SNS users who are afraid of being evaluated by others and do not want to be noticed disclose less personal information on their profile pages to protect themselves from harm and disappointment (Chen et al., 2019). In other words, individuals may reduce their amount of online self-disclosure as the simplest way to reduce the risks of wasting personal time and effort and damaging their image (Hwang et al., 2019).

Accordingly, we propose the following hypotheses:

H1a: FPE is negatively associated with the AOSD.
H1b: FNE is negatively associated with the AOSD.

Kim et al. (2015) found an inverse relationship between the AOSD and DOSD. Previous research has indicated that the frequency and number of posts can be reduced, but individuals still present their most attractive side as much as possible in infrequent posts (Leary and Allen, 2011; Rui and Stefanone, 2013; Hollenbaugh and Ferris, 2014). This finding indicates that although fear of evaluation may weaken users' willingness to disclose in terms of frequency and duration, it enhances users' motivation to make a deep or good impression on others (Lee and Jang, 2019). Those who are worried about being judged by others are willing to spend much time and energy creating a public image that they feel can be avoided from being misunderstood or even misinterpreted (Leary and Allen, 2011). Therefore, disclosers may maintain, modify or redeem their public image by revealing more information in-depth or information close to their true self.

Accordingly, we propose the following hypotheses:

H2a: FPE is positively associated with the DOSD.
H2b: FNE is positively associated with the DOSD.

### The Moderating Role of Protective Face Orientation

Face is closely related to traditional Chinese Confucianism (Tu, 1999), but the academic concept of “face” was first proposed by Goffman (1967). His concept of “face” or “facework” is defined as “a person (who) effectively declares his positive social value” and is a self-image depicted by recognized social attributes that is usually regarded as something that can be maintained or lost in interaction (Goffman, 1967). Brown and Levinson (1987) elaborated on this from the perspective of polite communication. For example, in Western culture, this phenomenon is mainly described in terms of “embarrassment,” “politeness,” “self-protection,” “respect,” and “obedience” and is reflected, for example, in the way individuals maintain face while building friendships on social media (Goffman, 1956; Thomas, 1992; Wood and Forest, 2016; Kwek et al., 2019; Ditchfield, 2020). Chinese people are particularly concerned about the opinions and comments of others and do not dare to reveal their true selves in public or in interpersonal communications because they are afraid of exposing their shortcomings, such as clumsiness, ignorance and incompetence; this exposure may result in the experience of losing face, including criticism, rejection and ridicule by others (Oetzel et al., 2001; Zane and Ku, 2014; Guan and Lee, 2017; Song, 2019). For example, due to the fear of losing face, Chinese students are more reluctant to ask for help in the community or ask questions in class than students in other countries (Leong et al., 1995; Hwang et al., 2003; Heath et al., 2016).

To avoid shame and losing face, individuals implement social strategies to protect themselves from social evaluation (Kamalou et al., 2019). PFO is a social psychological construction with roots in the Chinese context that has evolved within Chinese values and philosophy, constituting a unique characteristic of the country (Zhang et al., 2011; Chen et al., 2018; Lee and Hwang, 2019; Song, 2019). When face is threatened, PFO will activate a psychological defense mechanism and self-protection strategy (Chou, 1996; Hwang, 2006; Wang et al., 2016; Ramada, 2020), which not only stimulate their fear response to the evaluation of others but also increase their self-regulation behavioral concerns (Cupach and Carson, 2002; Vogel and Wester, 2003; Baumeister et al., 2005; Kamalou et al., 2019).

Individuals with higher PFO levels are more sensitive and concerned with praise, criticism, standards, and evaluations from the external environment and generally find it more difficult to withstand evaluations by others. If individuals with fear of evaluation have a high PFO, their sensitivity to others' opinions and evaluations may lead them to decide to hide and avoid when they encounter negative comments or positive praise; for example, due to fear of forming some type of weak or stupid stereotype in the eyes of others (Wang et al., 2016; Rui and Stefanone, 2018), such individuals avoid appearing in the public space by reducing the amount of self-disclosure (Lim et al., 2012; Lee, 2014; Zeng and Zhu, 2019). These individuals would rather refuse to disclose the interpersonal reciprocity introduced by disclosure and block their opportunities or possibilities to connect with others (Chen et al., 2017; Song, 2019). In addition, individuals with high PFO are often described as having “thin face” and being overly concerned with others' evaluation and standards (Murray, 1999; Chan, 2012; Charmaraman et al., 2018), resulting in a relatively weaker risk tolerance. In reality, it is difficult to guarantee that everything shared will always be recognized and appreciated (Rui and Stefanone, 2013). Therefore, when they are determined to reveal deeper, higher-quality content to rebuild or maintain their image, they may encounter individuals who judge them and express opinions that they do not like. However, these few comments can be enough to destroy their self-esteem and confidence. Therefore, due to their need for the long-term maintenance of face and their weak ability to cope with such uncertain risks, they are more likely to stop relying on high-quality disclosure to maintain their image and face.

**Figure 1:**
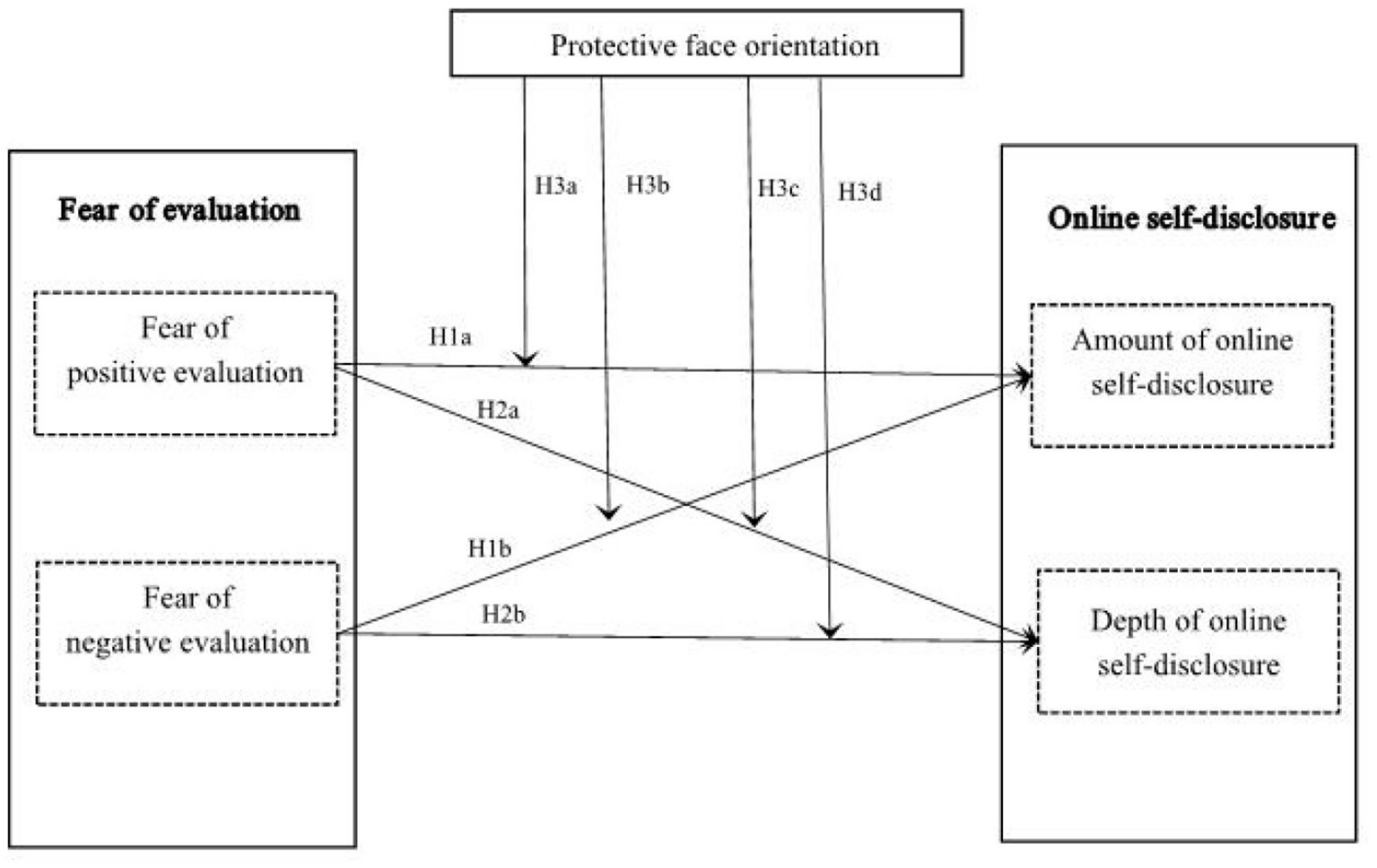
Theoretical model.

Accordingly, we propose the following hypotheses:

RThis study constructs a theoretical framework of users' online self-disclosure (see Figure 1).

## Research Design

### Participants

The participants included college students with experience using WeChat Moments who were recruited from universities located in College Town, Chongqing City. WeChat, a platform integrating instant messaging, voice chat, online shopping and mobile payment, was launched by Tencent in early 2011. WeChat has become the main social media platform on which Chinese netizens maintain interpersonal relationships and obtain social support (Wang et al., 2018). The platform has a function called WeChat Moments, which provides a mobile social networking service that enables users to freely share anything with others, such as personal identity updates or information. In addition, users can repost, like or comment on their friends' statuses.

There are 15 universities in College Town, including first-tier (two national universities), second-tier (six provincial universities) and third-tier (seven vocational colleges) institutions. To ensure the diversity of participants, the selected universities belonged to different tiers, with one from the first tier, two from the second tier and two from the third tier. We determined the number of students drawn from various schools according to the overall proportion of the number of college students in the above five universities. A total of 900 questionnaires were distributed and 832 questionnaires were collected, for a return rate of 92.44%. When we excluded invalid questionnaires, the effective response rate was 83.33% (*N* = 750).

The demographic characteristics (see Table 1) of the participants were as follows: 412 (54.93%) were male and 338 (45.07%) were female; 36.80% majored in engineering, 24.40% in social science, 15.33% in natural science, 15.73% in arts and humanities, and 7.73% in medicine. Most participants were undergraduates (44.00%), followed by vocational college students (29.60%), graduate students (23.07%) and doctoral students (3.33%). Most participants were aged between 18 and 26 years (98.00%). The data collection relied on respondents' self-assessments, which may exaggerate or understate the real research sample.

**Table 1:** Demographic profiles.

**Demographic** **variable**	**Item**	**Frequency**	**Percentage (%)**	**Cumulative** **percentage (%)**
Gender	Male	412	54.93	54.93
	Female	338	45.07	100
Age	18–20	260	34.67	34.67
	21–23	340	45.33	80.00
	24–26	135	18.00	98.00
	27–29	10	1.33	99.33
	≥30	5	0.67	100
Education	Vocational college students	222	29.6	29.6
	Undergraduates	330	44	73.6
	Graduate students	173	23.07	96.67
	Doctoral students	25	3.33	100
Major	Arts and humanities	118	15.73	15.73
	Social science	183	24.4	40.13
	Natural science	115	15.33	55.47
	Engineering	276	36.8	92.27
	Medicine	58	7.73	100
Total		750	100	100

### Measurement of Instruments

Five variables were measured in this study: FPE, FNE, PFO, the AOSD and the DOSD. The questionnaire was adapted from classic scales originally developed in English. These scales were translated into Chinese by a Chinese teacher with 1 year of experience studying in the United States and by two graduate students with high English proficiency. Without changing the overall structure of the scale, we made partial modifications to the translation to better reflect the Chinese context. Except for the demographic variables, all questions in the questionnaire were based on a 5-point Likert scale (1 = *strongly disagree*, 2 = *disagree*, 3 = *neutral*, 4 = *agree*, 5 = *strongly agree*). The Appendix contains all the measurement questions for each scale.

#### Fear of Evaluation

The Fear of Positive Evaluation Scale (FPES) was adapted from Weeks et al. (2008). Davoudi et al. (2012) reported that the convergent and discriminant validity of the FPES have good reliability (Cronbach's = 0.890). The measurement items adopted in this study included eight items, for which the Cronbach's alpha was 0.913 (M = 2.78, SD = 0.858, KMO = 0.913). The Brief Fear of Negative Evaluation Scale (BFNES) developed by Leary (1983) has 12 items, including four reverse-scored items. Some studies have found that these reverse-scored items could cause confusion and incorrect responses, and Weeks et al. (2008) suggested that its positive items were sufficient to effectively measure FNE. Therefore, the four reverse-scored items were dropped. The Cronbach's alpha for the eight remaining items was 0.903 (M = 3.03, SD = 0.906, KMO = 0.937).

#### Protective Face Orientation

The Protective Face Orientation Scale (PFOS) was adapted from Zhang et al. (2011). Since its introduction, this scale has been widely used to measure people's face and facework, particularly among Chinese participants. For example, it was further developed by Chen et al. (2018) and Wang et al. (2016). The scale contains a total of six items, for which the Cronbach's alpha was 0.821 (M = 3.18, SD = 0.741, KMO = 0.846).

#### Online Self-Disclosure

The Online Self-Disclosure Scale (OSDS) was slightly modified from Wheeless (1976) and Huang (2016). It is divided into the Amount of Online Self-Disclosure Scale (AOSDS) and the Depth of Online Self-Disclosure Scale (DOSDS). The AOSDS consists of four items, which were reverse-coded and obtained a Cronbach's alpha of 0.808 (M = 2.85, SD = 0.754, KMO = 0.745). The DOSDS consists of three items, for which the Cronbach's alpha was 0.885 (M = 2.89, SD = 0.969, KMO = 0.742).

### Statistical Technique

Before the formal test, the questionnaire was reviewed by several researchers who were doctoral candidates and WeChat users to verify its logical consistency, ease of understanding, wording, and appropriateness. Further modifications were made in response to their feedback. Finally, the questionnaire was distributed to the target participants. During the formal survey process, we informed each participant that the survey was completely anonymous, and survey links were sent via WeChat platforms. To encourage participation, each participant could participate in a lottery after completing the survey and could randomly obtain ¥10–300 as a reward in cash or an online English class.

This study used SPSS as a data analysis tool for descriptive statistics, correlation analysis, and regression analysis. We used Harman's single-factor test to test for common method bias (Podsakoff et al., 2003). Specifically, we loaded all items into an exploratory factor analysis. Component analysis both with and without rotation revealed seven components, which together explained 68.36% of the total variance. The first factor that emerged from the unrotated factor solution had an explanatory level of 33.25%, indicating that the common method bias in this research was acceptable.

## Research Results

### Descriptive Statistics and Correlation Analysis

The descriptive statistics and correlation analysis results for each study variable are shown in Table 2, including the mean (M), standard deviation (SD), FPE, FNE, PFO, AOSD, and DOSD. The correlation analysis showed significant negative correlations between FPE and AOSD (*r* = −0.229, *p* < 0.01) and FNE and AOSD (*r* = −0.213, *p* < 0.01), significant positive correlations between FPE and DOSD (*r* = 0.343, *p* < 0.01) and FNE and DOSD (*r* = 0.288, *p* < 0.01), a significant negative correlation between PFO and AOSD (*r* = −0.250, *p* < 0.01), and a significant positive correlation between PFO and DOSD (*r* = 0.295, *p* < 0.01).

**Table 2:** Descriptive statistics and correlation analyses for each research variable.

	**M**	**SD**	**FPE**	**FNE**	**PFO**	**AOSD**	**DOSD**
FPE	3.163	0.979	—				
FNE	3.328	0.979	0.731^**^	—			
PFO	3.488	0.833	0.405^**^	0.383^**^	—		
AOSD	2.515	0.926	−0.229^**^	−0.213^**^	−0.250^**^	—	
DOSD	3.059	1.076	0.343^**^	0.288^**^	0.295^**^	−0.287^**^	—

*Sample size = 750*;

** *p < 0.01*.

### Regression Analysis

Regression analysis was used to test the hypotheses. Before performing regression analysis, we centered all variables to reduce the multicollinearity of the regression equation. Tables 3, 4 show the results of the regression analysis of the AOSD and DOSD as the dependent variables. First, the control variables were included in the regression, and then FPE and FNE were included in the regression model (Models 1 and 2). The third step was to add PFO to the regression model (Models 3 and 4). In the fourth step, the interaction items of FPE and PFO and the interaction items of FNE and PFO were added to the regression model (Models 5 and 6). Models 7–12 were derived by simply replacing the dependent variable AOSD with DOSD.

**Table 3:** Hierarchical regression results for the amount of online self-disclosure.

**Variable**	**Dependent variable: amount of online self-disclosure**					
	**Model 1**	**Model 2**	**Model 3**	**Model 4**	**Model 5**	**Model 6**
**Constant**	1.975	1.918	2.010	1.974	2.000	1.946
**Control variables**						
Gender	0.141^*^	0.183^**^	0.140^*^	0.166^*^	0.129	0.159^*^
Age	0.195^**^	0.199^**^	0.176^**^	0.178^**^	0.162^**^	0.174^**^
Education	−0.016	−0.021	−0.015	−0.017	0.010	−0.014
**Independent variables**						
FPE	−0.176^**^		−0.113^**^		−0.117^**^	
FNE		−0.172^**^		−0.113^**^		−0.107^**^
**Moderating variables**						
PFO			−0.190^**^	−0.192^**^	−0.193^**^	−0.187^**^
**Interaction terms**						
FPE × PFO					0.137^**^	
FNE × PFO						0.122^**^
*F*	17.513^***^	17.410^***^	18.420^***^	18.487^***^	18.658^***^	14.855^***^
*R* ^2^	0.086	0.085	0.110	0.111	0.131	0.127
Adjusted *R*^2^	0.081	0.081	0.104	0.105	0.124	0.120
Δ*R*^2^	0.086	0.085	0.024	0.025	0.021	0.016

*Sample size = 750*;

* *p < 0.05*;

** *p < 0.01*;

*** *p < 0.001*.

**Table 4:** Hierarchical regression results for the depth of online self-disclosure.

**Variable**	**Dependent variable: depth of online self-disclosure**					
	**Model 7**	**Model 8**	**Model 9**	**Model 10**	**Model 11**	**Model 12**
**Constant**	3.285	3.427	3.243	3.348	3.252	3.367
**Control variables**						
Gender	0.040	−0.047	0.042	−0.024	0.052	−0.020
Age	−0.129^*^	−0.148^**^	−0.106^*^	−0.119^*^	−0.094	−0.117^*^
Education	−0.021	−0.010	−0.023	−0.015	−0.026	−0.017
**Independent variables**						
FPE	0.360^**^		0.284^***^		0.287^**^	
FNE		0.295^**^		0.213^**^		0.209^**^
**Moderating variables**						
PFO			0.231^***^	0.265^**^	0.234^**^	0.262^**^
**Interaction terms**						
FPE × PFO					−0.116^**^	
FNE × PFO						−0.082^*^
*F*	27.235^***^	19.892^***^	27.086^***^	22.596^***^	24.486^***^	19.704^***^
*R* ^2^	0.128	0.096	0.154	0.132	0.165	0.137
Adjusted *R*^2^	0.123	0.092	0.148	0.126	0.158	0.130
Δ*R*^2^	0.128	0.096	0.026	0.035	0.011	0.005

*Sample size = 750*;

* *p < 0.05*;

** *p < 0.01*;

*** *p < 0.001*.

#### Control Variables

Gender and age had a positive effect, indicating that females had more self-disclosures than males (*β* = 0.141, *p* < 0.05); and with an increase in age, the amount of self-disclosure increased (*β* = 0.195, *p* < 0.01). Age had a significant negative effect on DOSD (*β* = 0.129, *p* < 0.05), indicating that the depth of self-disclosure became weaker with age (Model 7).

#### Direct Effects of Fear of Evaluation and Online Self-Disclosure

According to the results of Model 1 and Model 2 in Table 3, FPE had a significant negative effect on the AOSD (*β* = −0.176, *p* < 0.01), as did FNE (*β* = −0.172, *p*<0.01). The results showed that the higher the levels of FPE and FNE were, the lower the AOSD, thus supporting H1a and H1b. According to the results of Model 7 and Model 8 in Table 4, FPE had a significant positive effect on the DOSD (*β* = 0.360, *p* < 0.01), and FNE had a significant positive effect on the DOSD (*β* = 0.295, *p* < 0.01). Thus, the higher the levels of FPE and FNE were, the greater the DOSD, thus supporting H2a and H2b. Therefore, a higher degree of fear of evaluation was associated with lower AOSD and higher DOSD.

#### Moderating Effect of PFO

As shown in Table 3, the coefficient of the interactive item in Model 5 was significant (*β* = 0.137, *p* < 0.01), indicating that PFO plays a moderating role in the relationship between FPE and AOSD. The coefficient of the interaction term in Model 6 was also significant (*β* = 0.122, *p* < 0.01), indicating that PFO also plays a moderating role in the relationship between FNE and AOSD. As shown in Table 4, the coefficient of the interaction item in Model 11 was significant (*β* = −0.116, *p* < 0.01), indicating that PFO plays a moderating role in the relationship between FPE and DOSD. The coefficient of the interaction term in Model 12 was also significant (*β* = −0.082, *p* < 0.05), indicating that PFO plays a moderating role in the relationship between FNE and DOSD.

**Figure 2:**
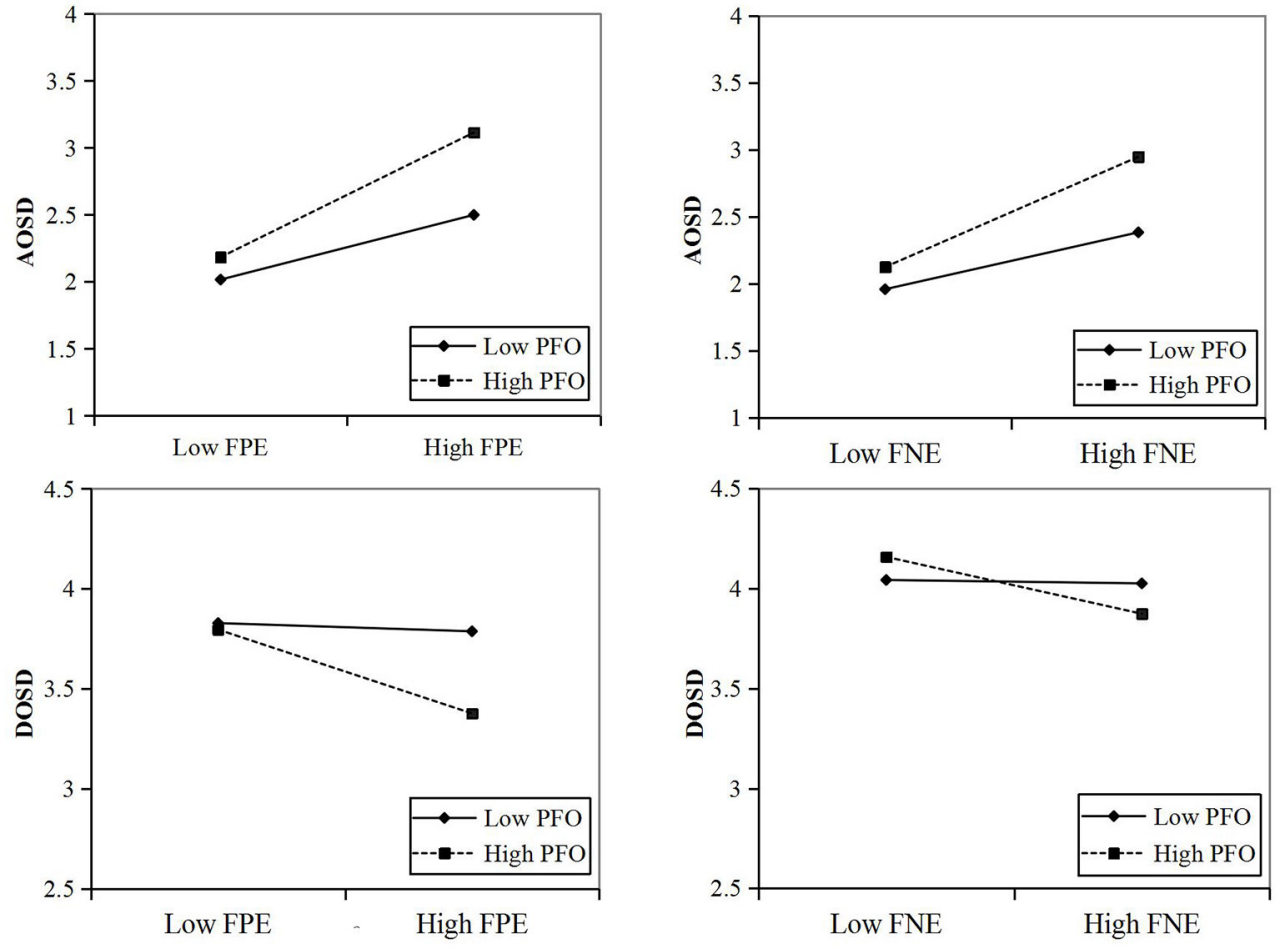
Moderating effect. PFO, protective face orientation; FPE, fear of positive evaluation; FNE, fear of negative evaluation; AOSD, amount of online self-disclosure; DOSD, depth of online self-disclosure.

To visually demonstrate the moderating effect, we performed a simple slope test. We divided PFO into two different levels, strong PFO (M + SD) and weak PFO (M – SD), to detect the specific moderating effects on the AOSD and DOSD, respectively; these interaction results are plotted in Figure 2. This finding shows that when PFO is stronger, FPE and FNE exert a greater impact on the AOSD, and FPE and FNE exert a weaker impact on the DOSD. Therefore, H3 was supported.

## Conclusion

This study explores how fear of evaluation affects online self-disclosure and examines how this relationship is moderated by protective face orientation in the Chinese context. In total, 750 Chinese WeChat users constituted the sample for a questionnaire-based analysis and regression analysis. The results showed that both fear of positive evaluation and fear of negative evaluation had a significant negative effect on the amount of online self-disclosure and a significant positive effect on the depth of online self-disclosure. This study also confirmed that a negative correlation exists between the depth and quantity of online self-disclosure. More importantly, this study extended existing research efforts by showing that protective face orientation moderates the association between fear of evaluation and online self-disclosure in the Chinese context, including the social context.

## Discussion

First, fear of evaluation has different effects on the AOSD and DOSD. Specifically, FPE and FNE reduce individuals' AOSD while increasing their DOSD. Previous studies have found that amount and depth were only used as partial reference indicators to measure the dimensions of self-disclosure (Hollenbaugh and Ferris, 2014; Chen et al., 2019), and few studies considered the possibility of differences or even opposite directions between the dimensions. However, Kim et al. (2015) demonstrated the influence of the strength of interpersonal relationships on online self-disclosure and found that there was a negative correlation between the depth and quantity of online self-disclosure. Therefore, our study confirmed the relationship between these dimensions.

To maintain their self-image on SNSs, individuals may reduce their chances of being judged by engaging in fewer social interactions. The phenomenon of social avoidance is similar to the research on debaters by Lin (2019), lack of presence on SNSs, and lack of caring or effort dedicated to social interactions with members of one's network. Interestingly, given limited resources of time and energy (Hwang et al., 2019), people usually adopt a principle of low cost and high efficiency to improve the quality of the content they disclose online to restore or reshape their self-image, such as decreasing the AOSD while increasing the DOSD. Consistent with the existing literature (Wood and Forest, 2016; Zhang et al., 2019), to lessen the sting of rejection, individuals with a fear of evaluation may perceive greater role stress and may manage their public image and regulate their behavior in interpersonal life more cautiously and strategically when receiving public attention. From the perspective of resource theory, a certain correlation may exist between the decrease in the amount of disclosure caused by the energy and time consumed by users and the quality of disclosure (Halbesleben et al., 2014). Research suggests that self-improvement during rehearsal and editing before formal self-presentation is a form of energy appropriation. Elaborate editing is a strategy of quantity reduction in pursuit of “perfect” content (Ditchfield, 2020). Therefore, in this trade-off relationship, we should focus on the purpose of emphasizing the core attributes of the individual after the shift of energy rather than the simple desire to reduce social interaction with others. This concept is also considered in the proposition by Zhang et al. (2019), who reported a positive communication result caused by moderately intense social pressure.

Second, when moderated by PFO, individuals' online self-disclosure behavior became more cautious and conservative. Specifically, on the one hand, PFO strengthened the impact of fear of evaluation on the amount of online self-disclosure. These results show that the fear of evaluation reduces the amount of online self-disclosure more obviously under the psychological effect of face-saving. On the other hand, PFO weakens the effect of fear of evaluation on the depth of online self-disclosure. Therefore, the fear of evaluation no longer motivates individuals to impress others or save face. The combination of social stress, anxiety of face and fear of evaluation is beyond the threshold individuals can actively cope with and address and is followed by more social avoidance and negative socializing. One possible explanation is that rather than pandering or catering to audience preferences, an individual may find it preferable to protect his or her face and avoid presenting too much information.

This possibility extends the research of Goffman (1955, 1956), who found that to maintain self-image, people not only avoid evaluation that could damage their self-image but also sometimes deliberately hide aspects that others might not like or appreciate. In the Chinese context, highlighting the importance of collaborative culture and maintaining harmonious coexistence are common sense and are associated with the fear of violating customs, social norms and conventions. Being excluded, despised or rejected by others is considered shameful (Hu, 1944; Hwang, 1987; Lee, 2014; Zane and Ku, 2014; Chen et al., 2017). Therefore, the existence and effects of PFO in daily life prevent people from taking risks to express themselves because they will experience the shame of losing face if they refuse to comply with conventional social norms. The appropriate, advisable method is thus to keep a low profile to avoid attracting much attention and evaluation (Hwang, 2006).

### Theoretical and Practical Implications

This study reveals the effect of fear of positive evaluation on online self-disclosure. Previous studies have suggested that negative evaluations could affect users' online social behavior (Leary and Allen, 2011; Chen et al., 2019), while ignoring positive evaluations, such as praise or appreciation, could also cause social pressure and social anxiety among individuals. Therefore, the contribution of this study is the provision of a new perspective for exploring the mechanism of online self-disclosure within the Chinese context and a perspective for future research.

This study also reveals the situational constraints on the effect of fear of evaluation on online self-disclosure. When confronted with face threats, people use defense as the mainstay instead of outputting and packaging themselves in an offensive manner; instead, individuals block fear from the source and regain face in a gentle and conservative manner with defense as the mainstay likely because the face culture in the Chinese context still carries the label of traditional Confucianism (Tu, 1999); when conflicts occur, people tend to compromise, give in and remain calm (Oetzel et al., 2001). This type of self-disclosure, which is almost abandoning the maintenance of personal settings, may also be caused by complex factors, such as social fatigue, complicated interpersonal relationships, and excessive maintenance costs (Hwang et al., 2019). Therefore, even if the psychology of face-saving awakens their determination to rebuild or repair their personal image (Lee and Jang, 2019), their response behavior appears somewhat half-heartedly.

### Limitations and Directions for Future Research

The limitations of this study must be mentioned. First, according to previous research results, face is a complex, multidimensional concept that involves acquisitive face orientation (AFO) as well as other and mutual face (Chou, 1996; Croucher et al., 2020). However, to focus on the social situation of fear of evaluation, this study only preliminarily explored the influence of PFO on the relationship between fear of evaluation and online self-disclosure. Therefore, future research is needed to determine the different results caused by other categories of face as well as the possibility of using PFO as a mediator rather than a moderator. Second, because WeChat is a community of acquaintances and strong relationships, future research may consider whether the characteristics of PFO on other social platforms are similar and compare the performance characteristics associated with face on different platforms. Third, our research sample may be affected by self-selection bias. The questionnaire was primarily designed to attract WeChat active users who were interested in this question, but other user groups, such as users who are less concerned and have the same FPE and FNE, were not included in our consideration.

## Data Availability Statement

All datasets generated for this study are included in the article/Supplementary Material.

## Ethics Statement

The studies involving human participants were reviewed and approved by Academic Committee of School of Journalism and Communication at Chongqing University. The patients/participants provided their written informed consent to participate in this study.

## Author Contributions

RZ contributed to the research idea, data analysis, and model. DZ contributed to the literature review and data analysis. All authors contributed to paper's drafting, editing and review.

## Conflict of Interest

The authors declare that the research was conducted in the absence of any commercial or financial relationships that could be construed as a potential conflict of interest.

## Publisher's Note

All claims expressed in this article are solely those of the authors and do not necessarily represent those of their affiliated organizations, or those of the publisher, the editors and the reviewers. Any product that may be evaluated in this article, or claim that may be made by its manufacturer, is not guaranteed or endorsed by the publisher.
