# Supplementary material for: Fear of Evaluation and Online Self-Disclosure on WeChat: Moderating Effects of Protective Face Orientation
Source: Front Psychol. 2021 Aug 25;12:530722. doi: 10.3389/fpsyg.2021.530722 (PMC8424039; doi:10.3389/fpsyg.2021.530722)
Supplement: Supplementary file 1 [file Table_1.docx]

**Appendix**

**Scales**

**The Fear of Positive Evaluation Scale (FPES)**

"I am uncomfortable exhibiting my talents to others, even if I think my talents will impress them"; "It would make me anxious to receive a compliment from someone that I am attracted to"; "I try to choose clothes that will give people a little impression of what I am like"; "I feel uneasy when I receive praise from authority figures"; "I would rather receive a compliment from someone when that person and I are alone than when in the presence of others"; "If I was doing something well in front of others, I would wonder whether I was doing 'too well'"; "I generally feel uncomfortable when people give me compliments"; "I do not like to be noticed when I am in public places, even if I feel as though I am being admired".

**The Fear of Negative Evaluation Scale (FNES)**

"I worry what other people think of me even when I know it does not make any difference"; "I am frequently afraid of other people noticing my shortcomings"; "I am afraid that others will not approve of me"; "I am afraid that people will find fault with me"; "When I am talking to someone, I worry about what they may be thinking of me"; "I am usually worried about the kind of impression I make"; "Sometimes I think I am too concerned with what other people think"; "I often worry that I will say or do the wrong things".

**The Protective Face Orientation Scale (PFOS)**

"I do my best to hide my weakness in front of others"; "I prefer to maintain a minimum level of dignity rather than gain face"; "I am cautious and try to avoid making mistakes"; "I do not like to talk a lot to avoid attracting others' attention"; "I seem to be more fearful of losing face than others"; "I seem to be conservative and less self-promoting when talking to others".

**The Amount of Online Self-disclosure Scale (AOSDS)**

"I do not often talk about myself in my WeChat Moments"; "My statements of my feelings are usually brief in my WeChat Moments"; "My conversation is the shortest when I am discussing myself in my WeChat Moments"; "Only infrequently do I express my personal beliefs and opinions in my WeChat Moments".

**The Depth of Online Self-Disclosure Scale (DOSDS)**

"I intimately disclose who I truly am openly and fully in my conversation in my WeChat Moments"; "Once I get started, my self-disclosures last a long time in my WeChat Moments"; "I typically reveal information about myself without intending to in my WeChat Moments".
